# Mechanical Performance and Parameter Sensitivity Analysis of 3D Braided Composites Joints

**DOI:** 10.1155/2014/476262

**Published:** 2014-07-08

**Authors:** Yue Wu, Bo Nan, Liang Chen

**Affiliations:** ^1^School of Civil Engineering, Harbin Institute of Technology, No. 77 Huanghe Road, Nangang District, Harbin 150090, China; ^2^CAPOL International Design Group, Shenzhen 518038, China

## Abstract

3D braided composite joints are the important components in CFRP truss, which have significant influence on the reliability and lightweight of structures. To investigate the mechanical performance of 3D braided composite joints, a numerical method based on the microscopic mechanics is put forward, the modeling technologies, including the material constants selection, element type, grid size, and the boundary conditions, are discussed in detail. Secondly, a method for determination of ultimate bearing capacity is established, which can consider the strength failure. Finally, the effect of load parameters, geometric parameters, and process parameters on the ultimate bearing capacity of joints is analyzed by the global sensitivity analysis method. The results show that the main pipe diameter thickness ratio *γ*, the main pipe diameter *D*, and the braided angle *α* are sensitive to the ultimate bearing capacity *N*.

## 1. Introduction

CFRP truss is composed by members and joints, and the composite joint is the most important component, where the force and deformation are very complex, moreover there exists phenomenon of stress concentration, which is the weak point of the loading process [1, 2]. According to statistics, there are 70% spacecraft structure damages that occurred at the connecting part [3, 4]. Therefore, in the analysis and optimization of composite structures, the key point is the prediction of the joint strength and the influence on each parameter.

According to forming process it can be divided into Molding joint, Winding joint, Layer-Molding joint, and 3D braided joint. The first three joints belong to the laminated structure, and the theoretical research and manufacturing process are relatively mature, and they are commonly used in the CFRP truss joints currently [5, 6]. But the laminated CFRP joint strength between layers is small, delamination defects occurs easily. While the 3D braided CFRP also has fiber in thickness direction, overcoming the weakness of delamination in the former three joints [7]. Therefore, the 3-D braided joints are important forms to study on in all kinds of CFRP truss joints.

Recently, researches on 3D braided joint are mainly concentrated on material constants and numerical simulation and experiment. Zheng et al., and so forth, [8] taking CFRP 3-D braided spherical joint as the research object, studied its damage mode under complex loads. C.-Y. Yang and H.-N. Yang [9] studied the bending stiffness on three connected specimens. Zheng et al. and so forth [10] analyzed the lug load capacity of 3D four-directional braided. Sun et al. [11] studied the carbon/epoxy 3D multidirectional braided tubular joints by finite element analysis. The current study provides numerical analysis methods, but most of them do not get tested, and no one do correlation analysis on the influence of the CFRP parameters in numerical simulation.

The mechanical properties of K type 3D braided joints and the influence of CFRP parameters were selected for research in this paper. Numerical analysis of K type 3D braided joint is studied firstly, verified through an existing test, and then various parameters were discussed including the effects of load parameters, geometric parameters, and material parameters on weighing the sensitivity impact of the joint ultimate bearing capacity.

## 2. Numerical Analysis Method Based on Micromechanics

### 2.1. Material Model

**Figure 1:**
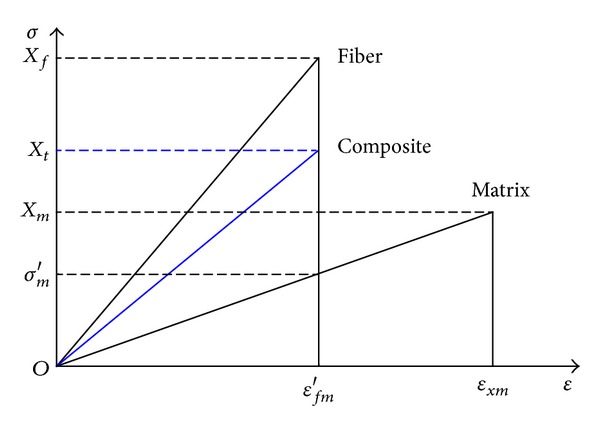
Stress strain curve of the fiber and matrix.

On the force along the fiber direction, Kelly and Davies put forward the hypothesis that all fibers have the same strength and more fragile than that of matrix (see Figure 1), if the composite has more than a minimum fiber volume content of *V*_*f*_, the composite is reaching its ultimate stress when the fiber deformation reaching its maximum strength. If the fiber strain along the fiber direction is equal to the matrix strain, the ultimate strength of the composite is

(1) $$\begin{array}{c} \sigma_{C\max}=\sigma_{f\max}V_{f}+{\left(\sigma_{m}\right)}_{\varepsilon_{f\max}}\left(1-V_{f}\right). \end{array}$$

In (1), *σ*_*C*max⁡_ is the ultimate stress of composite materials; *σ*_*f*max⁡_ is ultimate stress of fiber; and (*σ*_*m*_)_*ε*_*f*max⁡__ is the stress of ultimate matrix strain.

Because the fiber is brittle, it cannot exhibit elongation as matrix does. While the fiber damaged by longitudinal tension, composite material would be damaged, the strength of the composite is

(2) $$\begin{array}{c} X_{t}=X_{f}c_{f}+\sigma_{m}^{\prime }c_{m}. \end{array}$$

In (2), *X*_*t*_ is the fiber tensile strength; *σ*_*m*_′ is the matrix stress which is equal to the matrix stress when fiber tensile strain reached limit; *c*_*f*_ is cross-sectional area of the fiber; and *c*_*m*_ is the cross-sectional area of the matrix.

For the 3D braided joints, 3-cells model which was presented by De-long and Shen [12] is accepted in engineering in the prediction of elastic constants for 3D braided composite material. The basic idea is as follows.

(1) Calculate the elastic modulus of unidirectional composites.

Reference [12] gives the semiempirical formula which has been widely used in composite material area, as

(3) $$\begin{array}{c} E_{1}=E_{f1}V_{f}+E_{m}V_{m},\\ E_{2}=E_{3}=\frac{E_{m}}{1-\sqrt{V_{f}}\left(1-E_{m}/E_{f2}\right)},\\ G_{12}=G_{13}=\frac{G_{m}}{1-\sqrt{V_{f}}\left(1-G_{m}/G_{f12}\right)},\\ G_{23}=\frac{G_{m}}{1-\sqrt{V_{f}}\left(1-G_{m}/G_{f23}\right)},\\ \nu_{12}=\nu_{13}=\nu_{f12}V_{f}+\nu_{m}V_{m},\\ \nu_{23}=\frac{0.5E_{2}}{G_{23}}-1. \end{array}$$

In (3), *E*_1_, *E*_2_(*E*_3_); *G*_12_(*G*_13_), *G*_23_; *υ*_12_(*υ*_13_), *υ*_23_, respectively, are represented as the longitudinal elastic modulus, transverse elastic modulus; longitudinal shear modulus, transverse shear modulus; Poisson's ratio, transverse Poisson's ratio; *E*_*f*1_, *E*_*f*2_, *G*_*f*12_, *G*_*f*23_, *υ*_*f*12_ , respectively, are represented as the longitudinal elastic modulus of fiber, transverse elastic modulus, longitudinal shear modulus and Transverse shear modulus, longitudinal Poisson's ratio; *E*_*m*_, *G*_*m*_, *υ*_*m*_, respectively, are represented as elastic modulus, shear modulus, Poisson's ratio; *V*_*f*_ is fiber volume content and *V*_*m*_ = 1 − *V*_*f*_ is matrix volume content.

(2) Calculate the axial flexibility matrix of unidirectional composites.

Unidirectional composite axial flexibility matrix is shown in (4). Among them, *E*_1_, *E*_2_(*E*_3_), *G*_12_(*G*_13_), *G*_23_, *υ*_12_(*υ*_13_), *υ*_23_  are determined, respectively, by (3). Consider:

(4) $$\begin{array}{c} \left[S^{\prime }\right]=\left[\begin{array}{cccccc} \frac{1}{E_{1}} & -\frac{\nu_{12}}{E_{2}} & -\frac{\nu_{12}}{E_{2}} & 0 & 0 & 0\\ -\frac{\nu_{12}}{E_{2}} & \frac{1}{E_{2}} & -\frac{\nu_{23}}{E_{2}} & 0 & 0 & 0\\ -\frac{\nu_{12}}{E_{2}} & -\frac{\nu_{23}}{E_{2}} & \frac{1}{E_{3}} & 0 & 0 & 0\\ 0 & 0 & 0 & \frac{1}{G_{23}} & 0 & 0\\ 0 & 0 & 0 & 0 & \frac{1}{G_{12}} & 0\\ 0 & 0 & 0 & 0 & 0 & \frac{1}{G_{12}} \end{array}\right]. \end{array}$$

(3) Calculate the axial stiffness matrix of unidirectional composite

(5) $$\begin{array}{c} \left[C^{\prime }\right]={\left[S^{\prime }\right]}^{-1}. \end{array}$$

**Figure 2:**
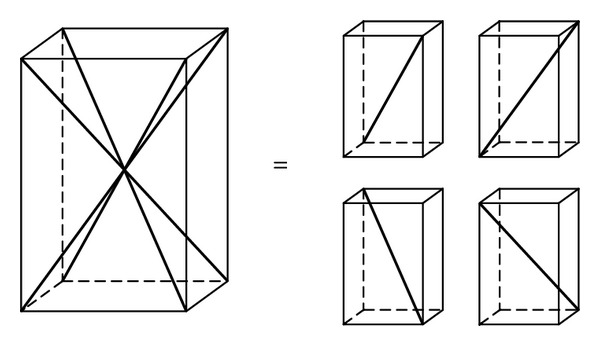
Internal cell and fiber (thick solid line) direction.

(4) Calculate transformation matrix of unidirectional composite from the local coordinate to global coordinate. The Internal cell and fiber direction are shown in Figure 2.

In order to obtain the unified material constants, the stiffness matrix in the local coordinates (*XYZ* coordinate) of unidirectional composites needs to be transformed into the global coordinate system. *X*′*Y*′*Z*′. Consider:

(6) $$\begin{array}{c} {\left[T\right]}_{i}=\left[\begin{array}{cccccc} l_{1}^{2} & m_{1}^{2} & n_{1}^{2} & 2m_{1}n_{1} & 2l_{1}n_{1} & 2m_{1}l_{1}\\ l_{2}^{2} & m_{2}^{2} & n_{2}^{2} & 2m_{2}n_{2} & 2l_{2}n_{2} & 2m_{2}l_{2}\\ l_{3}^{2} & m_{3}^{2} & n_{3}^{2} & 2m_{3}n_{3} & 2l_{3}n_{3} & 2m_{3}l_{3}\\ l_{2}l_{3} & m_{2}m_{3} & n_{2}n_{3} & m_{2}n_{3}+m_{3}n_{2} & n_{2}l_{3}+n_{3}l_{2} & l_{2}m_{3}+l_{3}m_{2}\\ l_{3}l_{1} & m_{3}m_{1} & n_{3}n_{1} & m_{3}n_{1}+m_{1}n_{3} & n_{3}l_{1}+n_{1}l_{3} & l_{3}m_{1}+l_{1}m_{3}\\ l_{1}l_{2} & m_{1}m_{2} & n_{1}n_{2} & m_{1}n_{2}+m_{2}n_{1} & n_{1}l_{2}+n_{2}l_{1} & l_{1}m_{2}+l_{2}m_{1} \end{array}\right], \end{array}$$

*l*_1_ = cos⁡*θ*, *l*_2_ = sin*θ*cos⁡*β*, *l*_3_ = sin*θ*sin*β*, *m*_2_ = sin*β*, *m*_3_ = −cos⁡*β*, *n*_1_ = −sin*θ*, *n*_2_ = cos⁡*θ*cos⁡*β*, *n*_3_ = cos⁡*θ*sin*β*.

**Figure 3:**
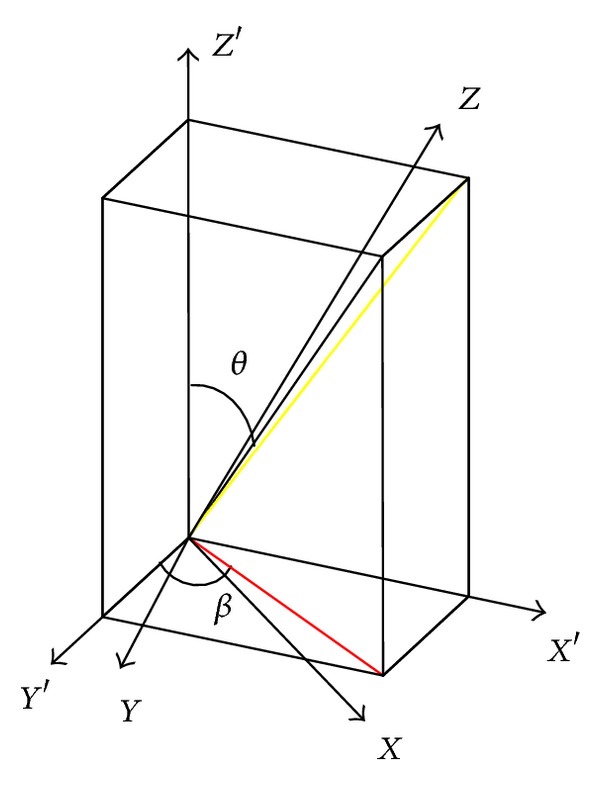
The coordinates of internal cell.

*θ*, *β* are defined as shown in Figure 3 (for *Z* coordinates of the fiber as an example): *θ* as the fiber angle between the projection of unit cell longitudinal plane and longitudinal axis, *β* as the fiber angle between the projection of unit cell transverse plane and horizontal axis, [*θ*, *β*] of the four fibers are [*θ*, 45°], [*θ*, −45°], [−*θ*, 45°], [−*θ*, −45°].

(5) Calculate the unidirectional composite stiffness matrix of partial axis equivalent in the global coordinate system

(7) $$\begin{array}{c} {\left[C\right]}_{i}={\left[T\right]}_{i}\left[C^{\prime }\right]{\left[T\right]}_{i}^{T}. \end{array}$$

(6) Calculate the unit cell overall stiffness matrix.

Unit cell overall stiffness is obtained by the average matrix stiffness of all fiber direction, and this method is called stiffness average method. Consider:

(8) $$\begin{array}{c} \left[C_{z}\right]=\frac{1}{4}\sum {\left[C\right]}_{i}. \end{array}$$

(7) Calculate the elastic constants of 3D braided composite materials by unit cell overall flexibility matrix.

3-cells model was used to predict the elastic constants of 3D braided composite material, which calculate stiffness matrix at each element according to the method which is shown in (3)–(8); then the whole stiffness matrix of the material could be calculated by stiffness matrix of each element according to the proportion of each unit cell by using the weighted sum method. Because of [13], unit cell stiffness matrix which occupies the largest proportion of the composite materials represents the stiffness matrix of braided material. Consider:

(9) $$\begin{array}{c} \left[S_{z}\right]={\left[C_{z}\right]}^{-1}. \end{array}$$

The elements of the flexibility matrix in whole unit cell are composed of elastic constants, as shown in (4). Therefore, the relationship between elastic constants of 3D braided composite material and the elements of unit cell overall flexibility matrix is as follows:

(10) $$\begin{array}{c} E_{x}=\frac{1}{S_{11}},\;\;E_{y}=\frac{1}{S_{22}},\;\;E_{z}=\frac{1}{S_{33}},\\ \;\;G_{yz}=\frac{1}{S_{44}},\;\;G_{zx}=\frac{1}{S_{55}},\;\;G_{xy}=\frac{1}{S_{66}},\\ \nu_{xy}=-\frac{S_{12}}{S_{22}},\;\;\nu_{yz}=-\frac{S_{23}}{S_{33}},\;\;\nu_{xz}=-\frac{S_{31}}{S_{11}}. \end{array}$$

*E*_*x*_, *E*_*y*_, *E*_*z*_ are the elastic modulus of 3D braided composites; *G*_*xy*_, *G*_*yz*_, *G*_*xz*_ are the shear modulus of 3D braided composites; *υ*_*xy*_, *υ*_*yz*_, *υ*_*xz*_ are the Poisson's ratio of 3D braided composites; *S*_*ij*_ is row *i* and column *j* in the overall flexibility matrix [*S*_*z*_].

After the MATLAB programming calculation, material elastic constants of this paper are *E*_*x*_ = 7.379e4 Mpa, *E*_*y*_ = *E*_*z*_ = 8.14e3 MPa, *G*_*xy*_ = *G*_*xz*_ = 1.256e4 MPa, *G*_*yz*_ = 4.03e3 MPa, and *υ*_*xy*_ = *υ*_*xz*_ = 0.8, *υ*_*yz*_ = 0.33. Test *E*_*x*_ differs by 4.15% from values in [14]. (Unified listed in Table 1.)

### 2.2. Geometric Analysis Model

**Figure 4:**
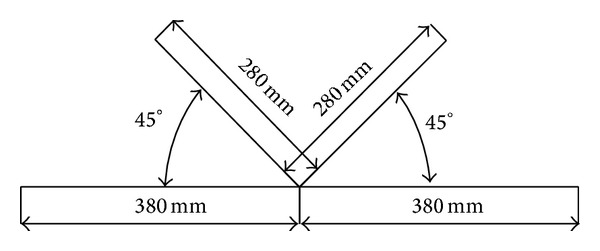
The size of K-joint.

In the K-joint size as shown in Figure 4, the diameter of main pipe is 120 mm, 4 mm of thickness; the diameter of branch pipe is 54 mm, 3.2 mm of thickness.

**Figure 5:**
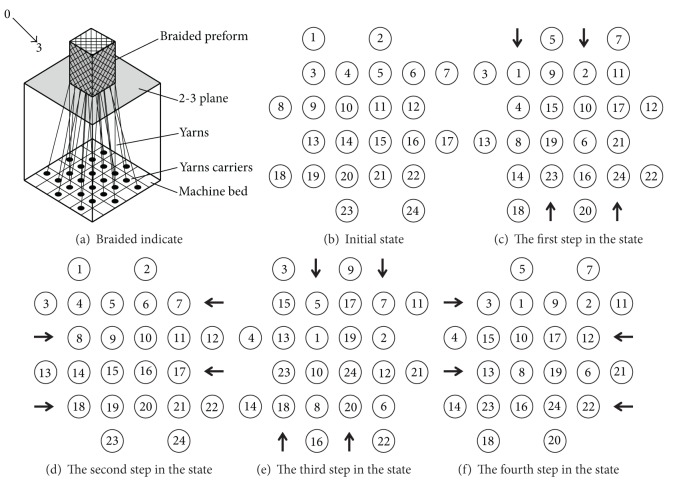
Four-step 1 × 1 3D braided process.

The K-joint adopts four step 1 × 1 (making one transverse and longitudinal movement for each time) woven technology (as shown in Figure 5), which braided angle is 25°, fiber volume content is 59%. Properties of the matrix and fiber (is shown in Table 1).

### 2.3. Unit Type and Mesh Density

#### 2.3.1. The Unit Type

SHELL93 and the SOLID92 were used to analyze the K-joint in this paper, the calculation results are shown in Table 2. It showed that the calculation results of 3D braided joint were less influenced by the type units. In order to improve the calculation efficiency, SHELL93 is used to analyze the K-joint in this paper.

#### 2.3.2. The Mesh Dividing

**Figure 6:**
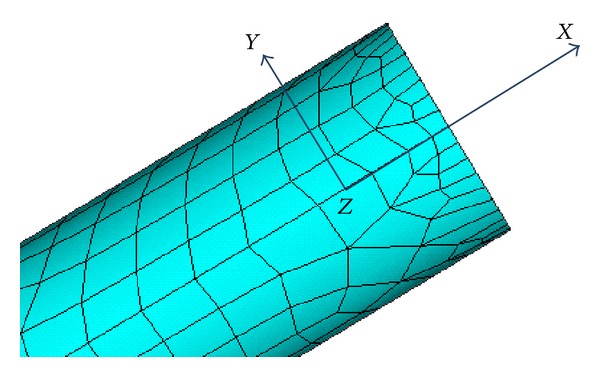
The unit coordinates of branch pipe.

Woven fibers have their own direction, branch and main pipe of the fiber direction differs, so as to overall performance. In order to simulate orthotropic character of material, the unit material properties should be corresponding to the fiber orientation. ANSYS provides the unit coordinate system to simulate the fiber direction, there are 2 ways: one is to mesh under different local coordinate; the other is to mesh together, then revise the unit coordinate to the local coordinate in unit character. The second method has been used in this paper, branch pipe divided unit coordinates is shown in Figure 6, in which the axial, tangential, and normal represent the unit coordinate three directions *X*, *Y*, *Z*.

The mesh precision has influence on the calculation results. The K-joints are divided along in five ways: sparse, slightly sparse, moderate, slightly dense, and dense, quantitative indicators which are shown in Table 3.

It can be seen that the joints ultimate bearing capacity differs 2% when grid is slightly dense compared with the densest, however, the error of joints' ultimate bearing capacity is larger when it divided sparsely. Considering the calculation of time and accuracy, the slightly dense grid is adopted in the paper.

### 2.4. Applying the Boundary Conditions and Load

**Figure 7:**
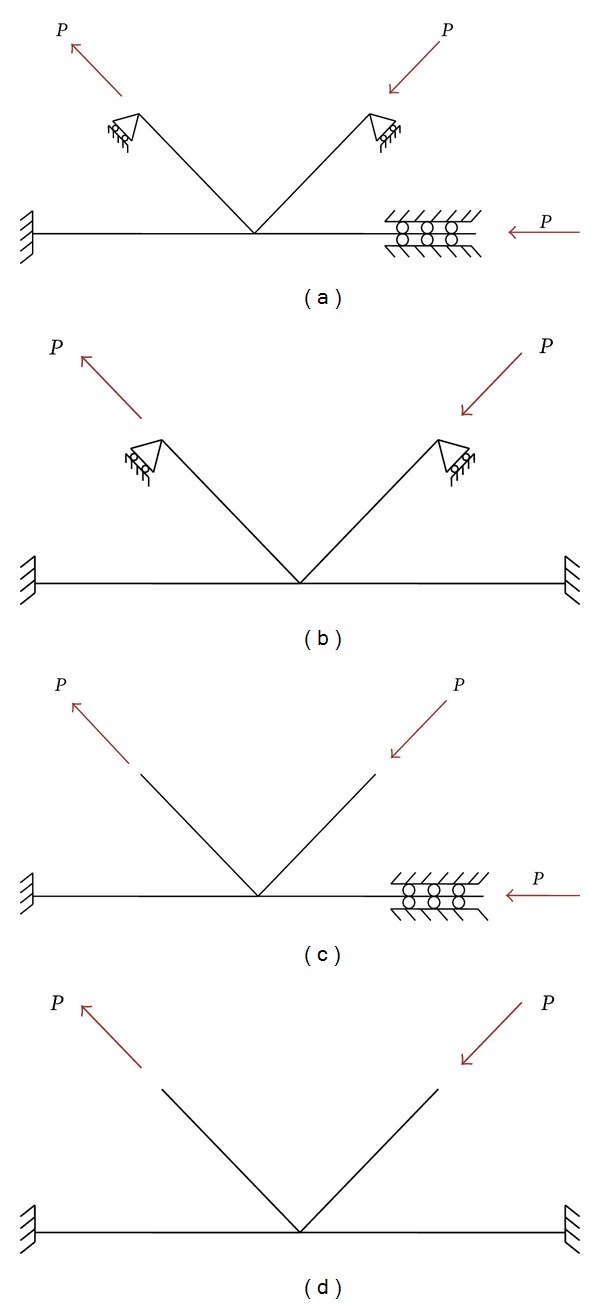
Boundary conditions and loading mode.

The common used boundary conditions and loading method in analysis of K-joint is shown in Figure 7. In (a) one end of the main pipe is fixed, the other is connected with the sliding hinge, the branch pipe is hinged; in (b) the main pipe is fixed at both ends, the branch pipe is hinged; in (c) one end of the main pipe is fixed, the other is connected with the sliding hinge, the branch pipe is free; in (d) the main pipe is fixed at both ends, the branch is free. When the main pipe is stressed, it becomes two-way loading, and is one-way loading when it does not stress.

**Figure 8:**
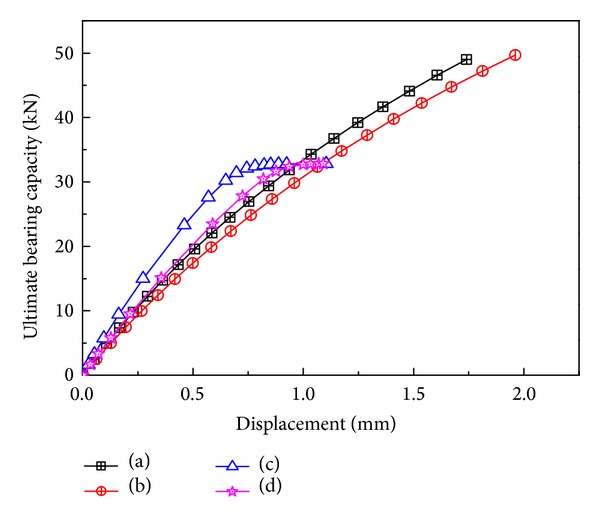
Loading-deformation curves in different boundary conditions.

When loaded in one-way direction, the Loading-deformation curves in different boundary conditions are shown in Figure 8. Constraint on (a) and (b), (c) and (d) the results are similar, which illustrates constraints of the joints are less influenced the ultimate bearing capacity; constraints on (a) and (c), (b) and (d) differed greatly, which illustrates constraints at end of the main pipe have less influence on the joints ultimate bearing capacity.

**Figure 9:**
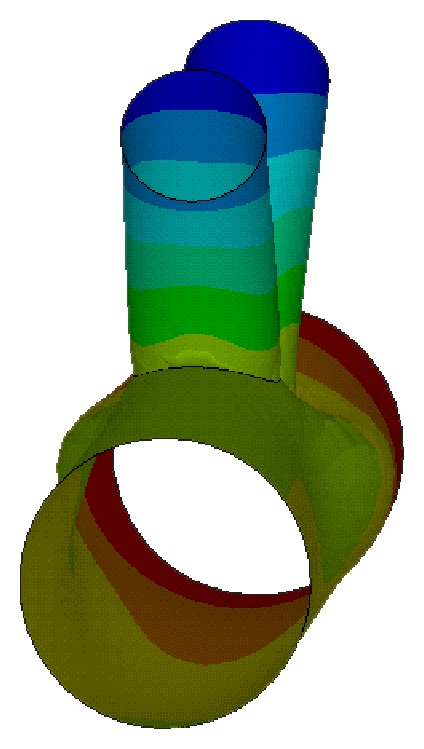
Plane deformation of branch pipe.

When the branch is free, the ultimate bearing capacity of the joints is pretty less than the branch pipe which is hinged; at the same time branch pipe of K-joint deformation is larger, which has deviated from the K-plane, as shown in Figure 9. Considering the actual structure of the branch pipe ends restrained by abdominal rod, the main pipe could move to some extent, therefore the boundary conditions of constraint (a) has been chosen in this paper, considering the one-way and two-way loading modes.

### 2.5. Failure Criterion and Ultimate Bearing Capacity

**Figure 10:**
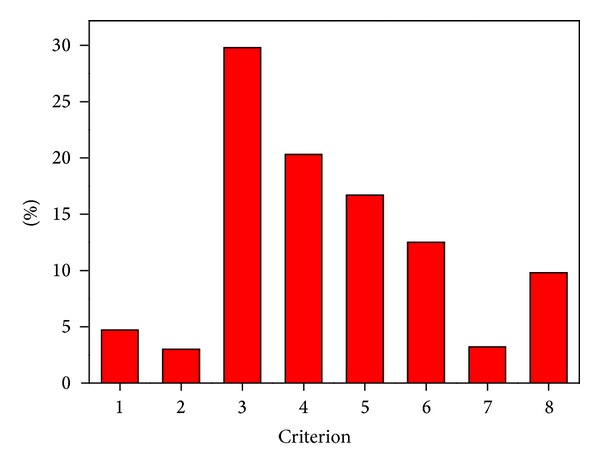
Frequency of application of composite material failure criteria. 1 Principal strain criterion; 2 strain energy density criterion; 3 the maximum strain criterion; 4 the maximum stress criterion; 5 Tsai-Hill criterion; 6 Tsai-Wu criterion; 7 strain-strain rate criterion; 8 other criterion.

Wang et al. [13] did statistic for usage of the composite materials failure criterion (as shown in Figure 10). The maximum stress criterion application frequency is ranked in second place, next to the maximum strain criterion. The maximum stress criterion was adopted in this paper, for this criterion is not only simple, practical, but also the test data of the maximum stress was verified in most of the reference for comparison.

*σ* is the maximum stress of Von. Mises of the joint, [*σ*] is the allowable material stress. [*σ*] is solved using Zuo [15] proposed 3D braided material strength of two order Tsai-Wu strength criterion based on prediction method and the maximum stress criterion. Prediction of the joint strength value is 610.7 MPa, the test value in the reference is 629 MPa, and the difference between the two is −2.9%.

When the maximum equivalent stress is higher than the material allowable stress, joints damage, and this load can be seen as the joint bearing capacity.

### 2.6. Numerical Methods for Verifying the Accuracy

**Figure 11:**
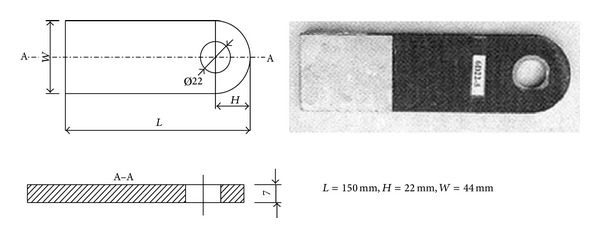
Single ear three-dimensional woven joint specimen and size.

The lug tensile test in [10] as shown in Figure 11. Carbon fiber material for the test is T700-12K, matrix material is TDE-85 epoxy resin, the braided angle is 20°, and fiber volume content is 45%. Calculation results show that the materials elastic constants are *E*_*x*_ = 7.198e4 MPa, *E*_*y*_ = *E*_*z*_ = 6.7e3 MPa, *G*_*yz*_ = 2.92e3 MPa, *G*_*xy*_ = *G*_*xz*_ = 7.81e4 MPa, *υ*_*xy*_ = *υ*_*xz*_ = 0.656, and *υ*_*yz*_ = 0.346. The material strength [*σ*] = 787.55 MPa.

**Figure 12:**
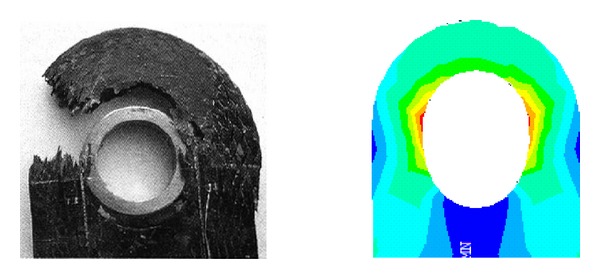
Lug joint failure pattern and the stress distribution.

3D braided joint numerical analysis method was used to make numerical simulation for the lug, calculating the ultimate bearing capacity which is 74.9 kN, testing value which is 79.4 kN, which differed 5.66%, the maximum stress position and joint breaking position are basically the same (as shown in Figure 12); that is to say, the numerical simulation method achieves certain precision, which can be used to simulate the 3D braided joint.

## 3. Analysis on the Parameter Sensitivity of 3D Braided Joint

After the analysis on the mechanical properties of 3D braided joint, it should make parameter sensitivity analysis on the parameters affecting the bearing capacity.

### 3.1. The Parameters and Value of 3D Braided Joint

In this paper, the effect of load parameters, geometric parameters, and process parameters on the ultimate bearing capacity of *N* was considered. The main parameters and their values are as shown in Table 4.

### 3.2. Analysis on the Global Parameter Sensitivity of 3D Braided Joint

According to whether to consider the interaction between parameters, parameter sensitivity analysis methods can be divided into local sensitivity analysis and global sensitivity analysis. Local sensitivity analysis refers to one parameter changed, and the other parameters remain unchanged, which can test the degree of influence on certain parameters variation to target results. Global sensitivity analysis refers to the influence on target results by changing one certain parameter when all the parameters changed. Because the global sensitivity analysis method considers the interaction between parameters, it is generally believed to be more accurate and scientific than the local sensitivity analysis method.

Since the parameter units of each node are not consistent, it needs to normalize the various parameters then make the parameters normalized to the range [0,1] by using the Equation: (*k* − *k*_min⁡_)/(*k*_max⁡_ − *k*_min⁡_) [16], to get the curve of normalized parameters and target results. The Equation is as follows:

(11) $$\begin{array}{c} S_{N}\left(k\right)=\frac{\left|\Delta N/N_{0}\right|}{\left|\Delta k\right|/\left(k_{\max}-k_{\min}\right)}, \end{array}$$

  *S*_*N*_(*k*)—sensitivity coefficient;  Δ*N*—variation target, taking the ultimate bearing capacity of *N* as a target;  *N*_0_—target reference value;  Δ*k*—variations of parameter *K*, and  *k*_max⁡_ − *k*_min⁡_—designed domain of parameter *K*.

Then it takes nonparametric statistical methods to make sensitivity analysis which is proposed by Marivoet and Saltelli in 1990. The method carried out regressing to analyze the parameters and the results, calculating the parameters and the standard variance and relative coefficient between the two, using the following equation to obtain sensitivity coefficients *S*_*i*_ under different parameters:

(12) $$\begin{array}{c} S_{i}=\frac{\mathrm{cov}\left(y,x_{i}\right)}{\sigma_{x_{i}}\sigma_{y}}, \end{array}$$

*S*_*i*_—coefficient parameter of parameter *x*_*i*_,  cov⁡(*y*, *x*_*i*_)—coefficient parameter of parameter *x*_*i*_, *σ*_*x*__*i*_—standard variance of parameter *x*_*i*_, and *σ*_*y*_—standard variance of parameter *x*_*i*_.

This paper selected *γ* = 30, *β* = 0.45, *θ* = 45, *τ* = 0.8, *n* = 0, *V*_*f*_ = 59%, *α* = 25°, and *d* = 120 mm as the joints reference state.

**Figure 13:**
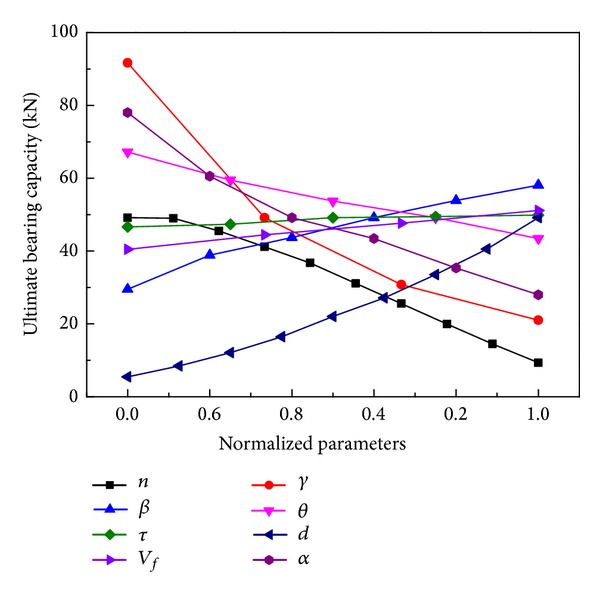
Relationship between ultimate bearing capacity and the normalized parameters.

Figure 13 shows an example of ultimate bearing capacity changed under different parameters. The slope of the curve is greater, the change of parameters has more influence on the ultimate bearing capacity, and the sensitivity is stronger.

Linear fitting for each parameter then take the average value, calculating sensitivity coefficient *S*_*N*_(*k*) in Table 5.

Equation (13) defined the parameter *K* (the relative contribution rate) as target sensitivity, that is, the ratio of either sensitivity coefficient or the sum of all the parameter sensitivity:

(13) $$\begin{array}{c} k=\frac{S_{N}\left(k\right)}{\sum S_{N}\left(k_{i}\right)}. \end{array}$$

**Figure 14:**
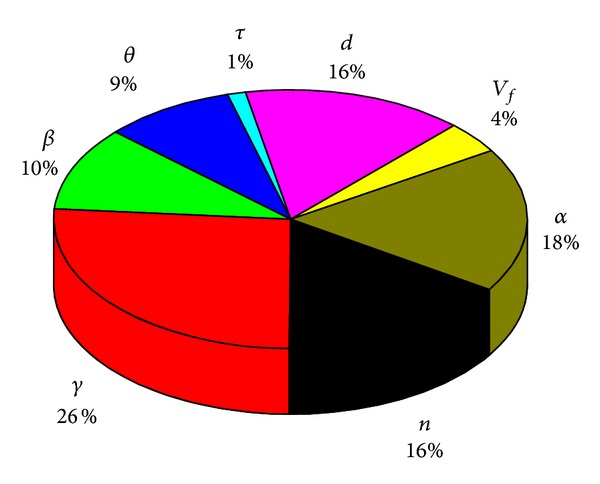
The relative contribution of various parameters on the sensitivity of *N*. *n*: the ratio of pipe force and material strength, *γ*: the ratio of main pipe diameter and thickness, *β*: the ratio of main pipe diameter and branch pipe diameter, *θ*: branch pipe axis and main pipe axis angle (°) *τ*: the ratio of main pipe wall thickness and branch pipe wall thickness, *α*: fiber braiding angle (°), *V*_*f*_: fiber volume content (%), and *d*: the main pipe diameter (mm).

According to the definition above, the relative contribution of various parameters on the sensitivity of *N* is the ratio of the sensitivity coefficient and the sum of all parameters sensitivity coefficient, which is shown in Figure 14.

## 4. The Conclusion

1. By studying the ultimate bearing capacity of 3D braided composite joint with numerical analysis method from the material model, element type, mesh size, boundary conditions, loads, and failure criteria six aspects, and compared the 3D braided composites with the lug, the numerical analysis method is proved feasible in this essay.
2. Based on these results, the main results about the sensitivity analysis of parameters on the 3D braided composite joint is as follows: the main truss diameter thickness ratio *γ*> fiber braiding angle *α*> load parameters *n*> competent diameter *d*> main and branch diameter ratio *β*> fiber volume content *V*_*f*_> branch and main thickness ratio *τ*. The ratio of thickness between the branch and main pipe has small influence on the ultimate bearing capacity.

## Figures and Tables

**Table 1:** Mechanical properties of T700 and TDE85.

The elastic constants	T700	TDE85
*E*_1_ (GPa)	221	3.4
*E*_2_ (GPa)	13.8	—
*G*_12_ (GPa)	9.0	—
*G*_23_ (GPa)	4.8	—
*υ*_12_	0.2	0.34
*υ*_23_	0.25	—
*X*_*ft*_ (MPa)	3528	85
*X*_*fc*_ (MPa)	2070	165
*ρ* (g/mm^3^)	1.77	1.21

**Table 2:** The influence of unit type on bearing capacity.

Unit type	Ultimate load (kN)	Relative error
Shell93	703	1.47%
Solid92	692.8	

**Table 3:** The influence of the mesh density on bearing capacity.

Mesh density	Number of element	Ultimate load (kN)	Relative error
Sparse	1039	54.34	12.65%
Slightly sparse	1520	50.64	4.98%
Moderate	2645	50.112	3.88%
Slightly dense	3685	49.21	2%
Dense	4031	48.24	—

**Table 4:** Joint parameters and the parameters values.

Parameter name	Parameter value				
Load parameter					
*n*	−0.9	−0.8	0	0.8	0.9
Geometric parameters					
*τ*	0.6	0.7	0.8	0.9	1
*γ*	20	30	40	50	—
*β*	0.35	0.4	0.45	0.5	0.55
*θ*	30	35	40	45	50
*d*	40	60	80	100	120
Process parameters					
*V*_*f*_	35	45	55	65	—
*α*	5	15	25	35	45, 55

*n*: The ratio of pipe force and material strength.

*γ*: The ratio of main pipe diameter and thickness.

*β*: The ratio of main pipe diameter and branch pipe diameter.

*θ*: Branch pipe axis and main pipe axis angle (°).

*τ*: The ratio of main pipe wall thickness and branch pipe wall thickness.

*α*: Fiber braiding angle (°).

*V*_*f*_: Fiber volume content (%).

*d*: The main pipe diameter (mm).

**Table 5:** The sensitivity coefficients of parameters on the *N*.

	*n*	*τ*	*γ*	*β*	*θ*	*α*	*V*_*f*_	*d*
*S*_*N*_(*k*)	0.9	0.1	1.4	0.6	0.5	1	0.2	0.8
